# The genetic basis of plasmid tropism between *Chlamydia trachomatis* and *Chlamydia muridarum*

**DOI:** 10.1111/2049-632X.12175

**Published:** 2014-05-08

**Authors:** Yibing Wang, Lesley T Cutcliffe, Rachel J Skilton, Kyle H Ramsey, Nicholas R Thomson, Ian N Clarke

**Affiliations:** 1 Molecular Microbiology Group, Faculty of Medicine, University of Southampton, Southampton General HospitalSouthampton, UK; 2 Microbiology and Immunology Department, Chicago College of Osteopathic Medicine, 10 Midwestern UniversityDowners Grove, IL, USA; 3 Department of Pathogen Genomics, The Wellcome Trust Sanger Institute, Wellcome Trust Genome CampusCambridge, UK

**Keywords:** plasmid, *Chlamydia*, tropism, transformation, replication

## Abstract

The development of genetic transformation technology for *Chlamydia trachomatis* using its endogenous plasmid has recently been described. *Chlamydia muridarum* cannot be transformed by the *C. trachomatis* plasmid, indicating a barrier between chlamydial species. To determine which regions of the plasmid conferred the species specificity, we used the novel approach of transforming wild-type *C. muridarum* carrying the endogenous plasmid pNigg and forced recombination with the *C. trachomatis* vector pGFP::SW2 which carries the complete *C. trachomatis* plasmid (pSW2). Penicillin and chloramphenicol-resistant transformants expressing the green fluorescent protein were selected. Recovery of plasmids from these transformants showed they were recombinants. The differences between the pSW2 and pNigg allowed identification of the recombination breakpoints and showed that pGFP::SW2 had exchanged a ∼ 1 kbp region with pNigg covering CDS 2. The recombinant plasmid (pSW2NiggCDS2) is maintained under antibiotic selection when transformed into plasmid-cured *C. muridarum*. The ability to select for recombinants in *C. muridarum* shows that the barrier is not at transformation, but at the level of plasmid replication or maintenance. Our studies show that CDS 2, together with adjoining sequences, is the main determinant of plasmid tropism.

The newly developed technique of genetic transformation for *Chlamydia* is set to significantly change the experimental approach to understanding more about this important pathogen. This manuscript adds critical new information in relation to the barriers to genetic transformation and shows, quite unexpectedly, that the cross-species barriers are actually replication-mediated tropisms, rather than transformation per se.

There are nine recognised species of *Chlamydia* each with distinctive biological properties such as a specific tissue tropism and disease pathology. Some new candidate species have recently been added to this list (Sachse *et al*., 2014). However, all the chlamydial species share the property of intracellular parasitism and grow within a modified cytoplasmic organelle (known as an inclusion) (Stephens *et al*., 2009). *Chlamydia* have a unique, biphasic developmental cycle alternating between an infectious elementary body (EB) and a replicating metabolically active form, the reticulate body (RB) (Ward, 1983). *Chlamydia trachomatis* is the leading worldwide infectious cause of blindness (trachoma), and genital chlamydial infection is the commonest diagnosed bacterial sexually transmitted infection (STI) in the Western world (Thylefors *et al*., 1995; Burstein & Zenilman, 1999). Infectious model systems have been set up with various animals (including nonhuman primates) using *C. trachomatis* to mimic human infections, but none of these accurately reflect natural human disease (Miyairi *et al*., 2010). A separate species *Chlamydia muridarum* causes respiratory tract infection in rodents and is the most well-studied, homologous (pathogen/host) small animal infection system. Thus, there is a great deal of interest in translating findings from the *C. muridarum* systems to understanding human genital and eye disease caused by *C. trachomatis*.

Almost all strains of *C. trachomatis* carry an endogenous 7500 bp plasmid; only four viable naturally occurring plasmid-free clinical isolates have been described; thus, these are exceedingly rare (Peterson *et al*., 1990; Farencena *et al*., 1997; Stothard *et al*., 1998; Wang *et al*., 2013a). Studies from plasmid-cured (where the plasmid has been physically removed by chemical agents) and naturally occurring plasmid-free chlamydia have shown that the presence of the plasmid is associated with the ability to accumulate glycogen, TLR2 activation and infectivity (O'Connell & Nicks, 2006; O'Connell *et al*., 2011; Russell *et al*., 2011). *In vivo* studies using a murine model and naturally occurring plasmid-deficient human genital *C*. *trachomatis* have shown that the plasmid is a virulence factor (Sigar *et al*., 2014). Preliminary experiments, with limited numbers of subjects, have indicated that a plasmid-cured human trachoma isolate of *C. trachomatis* is avirulent in the monkey eye and that this plasmid-cured isolate can elicit protective immune responses against the wild-type, plasmid-bearing strain (Kari *et al*., 2011).

Taken together, these studies indicate that the plasmid is a key determinant of virulence in both *C. trachomatis* and *C. muridarum,* and thus, there is renewed interest in understanding both its biochemical function and biological role. Eight major coding sequences (CDS) (> 100 bases) have been assigned to the chlamydial plasmid (Thomas *et al*., 1997). Antibodies specific to the predicted protein products encoded by each coding sequence (CDS) have been used to investigate the expression profile of these genes (Li *et al*., 2008). All are expressed during the developmental cycle, and the protein ‘pgp3’ encoded by CDS 5 is secreted beyond the inclusion and into the cell cytoplasm.

Recently, we developed a plasmid-based gene transfer system for *C. trachomatis* (Wang *et al*., 2011). This has proved useful in preliminary studies aimed at defining the biological function of several plasmid coding sequences (CDSs) and their protein products. Studies using natural mutants and employing this technology to make simple gene deletion and/or gene inactivations have shown that several plasmid genes are essential and likely have a role in plasmid maintenance. A focus of work on potential virulence factors has been the protein pgp4 (encoded by CDS 6), which is proposed as a ‘transcriptional regulator’, and the proteins encoded by CDS 7 (pgp5) and CDS 5 (pgp3) genes are dispensable for growth *in vitro* (Gong *et al*., 2013; Song *et al*., 2013; Wang *et al*., 2013a). Transformation studies have now been extended to *C. muridarum* (Song *et al*., 2014*)*. *Chlamydia muridarum* cannot be transformed by a *C. trachomatis* plasmid and *vice versa,* and this has been cited as an example of ‘transformation tropism’ (Song *et al*., 2014). The genetic basis for this plasmid-mediated, apparent tropism is unclear. To investigate the barriers to plasmid transformation between these chlamydial species, we cured *C. muridarum* (strain Nigg) of its plasmid using novobiocin as described previously (O'Connell & Nicks, 2006). The plasmid-free *C. muridarum,* designated *C. muridarum* Nigg P- (Plasmid minus), was purified by three rounds of plaque purification and confirmed to be plasmid free by PCR (data not shown). Repeated attempts to transform *C. muridarum* Nigg P- as the recipient host with the *C. trachomatis/Escherichia coli* plasmid shuttle vector pGFP::SW2 were unsuccessful in our hands, consistent with previous observations (Song *et al*., 2014).

To attempt to generate recombinants of pGFP::SW2 *in vivo* which can replicate in *C. muridarum,* we transformed wild-type, plasmid-bearing *C. muridarum* Nigg P+ with the *C. trachomatis* plasmid vector pGFP::SW2 (Wang *et al*., 2011). This encodes the GFP, *bla* and *cat* genes allowing expression of the green fluorescent protein and conferring resistance to penicillin or chloramphenicol, respectively. The transformation protocol was as previously described (Wang *et al*., 2011) except prolonged passage under penicillin (10 units mL^−1^) selection was used. After more than 2 weeks under penicillin selection (four passages), resistant inclusions emerged, but only a small portion of inclusions expressed green fluorescence. At this point, chloramphenicol (0.4 μg mL^−1^) was applied for selection as recently described (Xu *et al*., 2013), and after four rounds of chloramphenicol selection, almost all inclusions fluoresced green. Green fluorescent, penicillin and chloramphenicol-resistant *C. muridarum* were expanded by multiple passaging and a stock of bacteria produced. Whole DNA extracted from these *C. muridarum* transformants was used to transform *E. coli* to rescue ampicillin-resistant plasmids from the transformed *C. muridarum*. A total of 52 colonies expressing the green fluorescent protein were selected for plasmid DNA extraction. Fifty of the clones displayed the same Bgl II and/or Sal I restriction endonuclease digestion patterns. One of these was selected for sequence analysis and named pSW2NiggCDS2 (Fig.1a).

**Figure 1:**
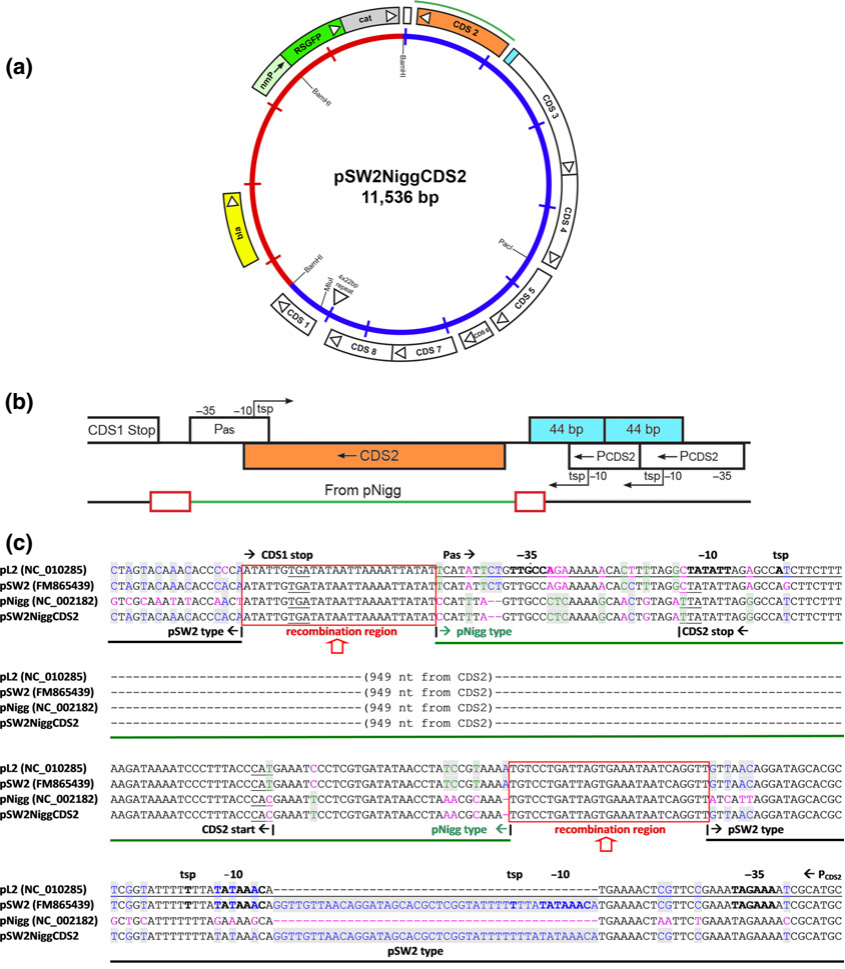
The structure of the recombinant plasmid pSW2NiggCDS2. (a) The whole plasmid map. (b) The CDS 2 region and flanking features. (c) The alignment of the sequence (in the region shown in b) with plasmids pL2, pSW2 and pNigg (GenBank accession numbers in brackets). The sequence from pNigg is represented by the green line, and the areas where recombination occurred are indicated by red boxes. P_as_, antisense promoter; P_CDS2_, CDS2 promoter; 44 bp, the characteristic 44 bp tandem repeat in pSW2. The start codon for CDS 2 and the stop codons of CDS 1 and CDS 2 are underlined in all the sequences. The promoters (P_as_ and P_CDS2_) are underlined only in the pL2 sequence. The −35, −10 and tsp features are highlighted in BOLD in pL2 and pSW2 (Ricci *et al*., 1995).

Plasmid pSW2NiggCDS2 is 11 536 bp in size. Sequencing showed that pSW2NiggCDS2 carries a minimum of 1055 bp originating from the plasmid pNigg replacing the equivalent 1058 bp from pGFP::SW2 (it is not possible to map the exact recombinational breakpoint as the sequences are identical for short stretches, see Fig.1c). Thus, for the productive replication of plasmid pGFP::SW2 in *C. muridarum,* the *C. trachomatis* progenitor plasmid's entire CDS 2, with some short flanking regions was replaced by the orthologous region from the *C. muridarum* plasmid pNigg. The replacement pNigg sequence started from the antisense promoter (P_as_) and ended at ∼ 20 bp before the unique 2 × 44 bp repeat in pSW2, and included the complete coding sequence for CDS 2. The recombinational breakpoints are located within the 26 bp identical sequences covering the CDS 1 stop codon and the 27 bp (or 30 bp) identical sequences covering the start of the first characteristic 44 bp tandem repeat, which is immediately upstream of CDS 3 (Fig.1b and c). To confirm whether the other clones were identical, we chose a further five clones and sequenced the CDS 2 region and found they were all exactly the same as pSW2NiggCDS2.

Our data build on recent work (Song *et al*., 2014) who reported that *C. trachomatis* plasmids could not be used to transform *C. muridarum*. Our results are consistent with these findings in which no transformants were observed when we attempted to transform *C*. *muridarum* Nigg P- with plasmid pGFP::SW2. For experimental rigour, it was necessary to show that recombinant *C. trachomatis* plasmid pGFP::SW2 with the replacement of the *C. muridarum* CDS 2 region (i.e. pSW2NiggCDS2) replicates in a *C. muridarum* background. Thus, we transformed plasmid-free *C. muridarum* Nigg P- with pSW2NiggCDS2. The transformants were rapidly recovered (within a week) under 10 units mL^−1^ of penicillin selection, and green fluorescent inclusions were visible at passage 3. Transformants were propagated for ten passages with penicillin selection. From this stock, transformants were grown with or without penicillin for a further five passages, and there were always some ‘nonfluorescent’ inclusions under both conditions. Figure2c and d show these transformants grown without penicillin, some of inclusions display the characteristic empty-centre or bull's eye phenotype under phase contrast microscopy (compare morphology with the parental host in Fig.2b), these inclusions do not fluoresce green. Figure2e and f show the transformants under constant penicillin selection; aberrant inclusions characteristic of sensitivity to penicillin through retardation of the developmental cycle occur (arrowed in Fig.2e); these inclusions do not fluoresce green. These results indicate that the loss of the transforming plasmid occurred in the presence or absence of selection with penicillin.

**Figure 2:**
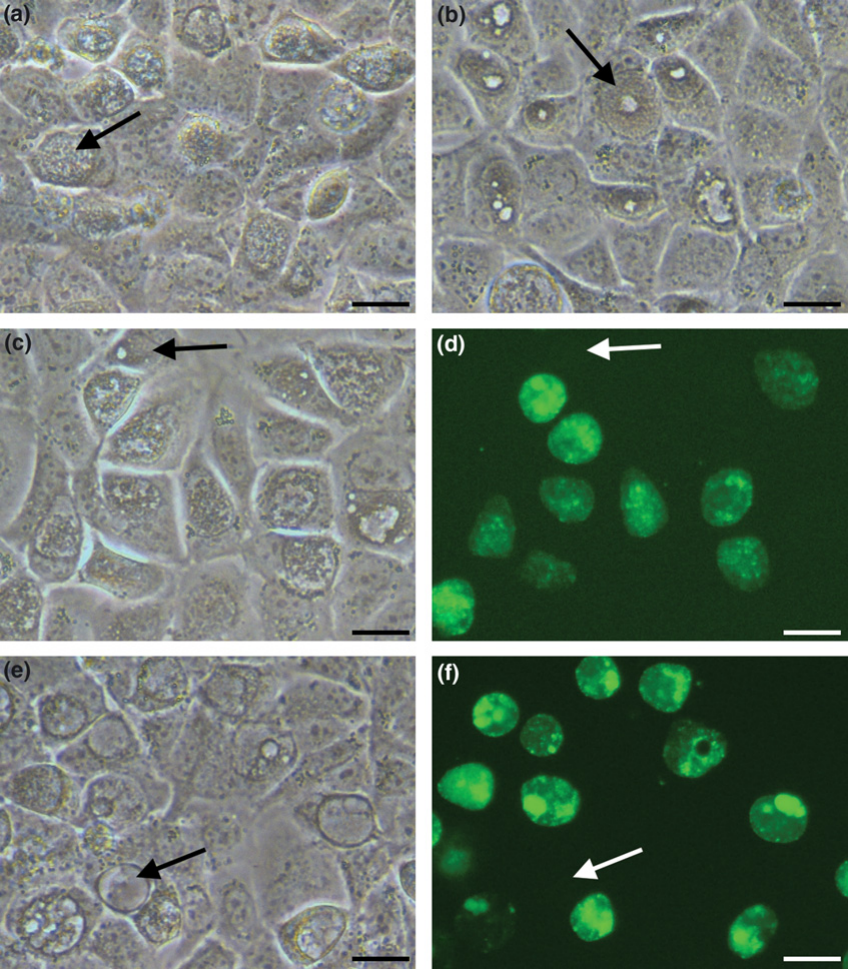
Images of *Chlamydia muridarum* infected McCoy cells at 28hpi. (a) Wild-type, plasmid-bearing *C. muridarum* (Nigg P+); the arrow shows a typical inclusion. (b) Plasmid-cured *C. muridarum* Nigg strain (Nigg P-, without plasmid); the arrow shows an inclusion with the distinctive plasmid-free bull's eye phenotype (i.e. ‘bright hole’ in the centre). (c–f) Transformants of *C. muridarum* Nigg P- with plasmid pSW2NiggCDS2, either without penicillin selection (c & d, same field), or under continuous penicillin selection (e & f, same field). When the transformants were grown without penicillin selection, some inclusions displayed the distinctive Nigg P- phenotype (an example is arrowed, (c), and they did not fluoresce green (d, arrowed). When the transformants were grown under penicillin selection, some inclusions displayed the distinctive aberrant inclusion phenotype of penicillin sensitivity (an example is arrowed in (e), and they did not fluoresce green (f, arrowed). (Scale bar = 20 μm)

The ability to select for recombinants in *C*. *muridarum* shows conclusively that the barrier is not at the point of transformation, but at the level of plasmid replication or maintenance. Our studies show that CDS 2, together with adjoining sequences (see Fig.1c), are the main determinants of plasmid tropism between *C. trachomatis* and *C. muridarum*. The low level of spontaneous loss of the plasmid pSW2NiggCDS2 on passaging suggests that these recombinants are less stable than the wild-type pNigg plasmid in *C. muridarum,* indicating other factors are also likely to contribute to plasmid retention, although CDS 2 is the main factor. Spontaneous plasmid loss occurs in *C. trachomatis* during culture (Matsumoto *et al*., 1998); this phenomenon has not been studied in detail in *C. muridarum*.

The cross-species barriers are actually replication-mediated tropisms rather than transformation tropism *per se*. Regardless, their presence has been confirmed by our results. By contrast to the report of ‘transformation tropism’ between *C. trachomatis* biovars (Song *et al*., 2014), our shuttle vector pGFP::SW2 (a genital tract based plasmid) can be used to transform LGV biovar isolates. Looking at the sequence of CDS 2 across the *C. trachomatis* biovars, it is evident that this is the most highly conserved gene of all the plasmid sequences, with only a single nonsynonymous SNP between them (Seth-Smith *et al*., 2009). Moreover, within the plasmid of *C. trachomatis,* the protein encoded by CDS 2 is not required for plasmid replication (Gong *et al*., 2013). Given the very high levels of sequence conservation, it seems unlikely that the CDS 2 region would determine *C. trachomatis* biovar ‘transformation tropism’. Thus, we need to seek an alternative explanation for the determinants of the tropism for *C. trachomatis* plasmids across different *C. trachomatis* biovars. Phylogeny has revealed high levels of diversity as well as the existence of recombinants within biovars, and natural recombinants between LGVs and non-LGV *C. trachomatis* have also been described (Jeffrey *et al*., 2013). The newly defined and distinctive clades T1 and T2 also encompass ocular and urogenital isolates (Harris *et al*., 2012); thus, barriers to plasmid replication between *C. trachomatis* biovars (Song *et al*., 2014) may not be biovar-specific, but strain-specific. The explanations for varying abilities to transform plasmids into *C. trachomatis* biovars may lie within the limits of the transformation technique itself (low transformation frequency and large vector, rendering definitions of ‘failure to transform’ unsatisfactory, especially as these are subjective rather than quantitative measures). Alternatively, the explanations may reside in the intrinsic properties of individual plasmids chosen for the studies. In this respect, our results may be unique for transformation of LGV isolates with the shuttle vector pGFP::SW2 (Wang *et al*., 2011, 2013a, b). The pSW2 plasmid is from the Swedish new variant of *C. trachomatis,* and this plasmid has two distinct features: a 377 bp deletion within CDS 1 and duplication at the 5′ end of CDS 3 (Seth-Smith *et al*., 2009). These features precisely flank the segment of DNA that has recombined from the endogenous *C. muridarum* plasmid pNigg to form pSW2NiggCDS2. The 377 bp deletion appears to inactivate CDS 1. The 44 bp repeated sequence at the 5′ end of CDS 3 duplicates the transcriptional start point (tsp) for CDS 2 (which is transcribed in the opposite direction) (Ricci *et al*., 1995; Albrecht *et al*., 2010). It is thus intriguing to hypothesise that the unique 44 bp duplication in the plasmid from the Swedish new variant is a favourable mutation that may have a significant biological role in conferring greater potential promiscuity to the wild-type plasmid pSW2 amongst *C. trachomatis*.

## References

[b1] Albrecht M, Sharma CM, Reinhardt R, Vogel J, Rudel T (2010). Deep sequencing-based discovery of the *Chlamydia trachomatis* transcriptome. Nucleic Acids Res.

[b2] Burstein GR, Zenilman JM (1999). Nongonococcal urethritis - a new paradigm. Clin Infect Dis.

[b3] Farencena A, Comanducci M, Donati M, Ratti G, Cevenini R (1997). Characterization of a new isolate of *Chlamydia trachomatis* which lacks the common plasmid and has properties of Biovar trachoma. Infect Immun.

[b4] Gong S, Yang Z, Lei L, Shen L, Zhong G (2013). Characterization of *Chlamydia trachomatis* plasmid-encoded open reading frames. J Bacteriol.

[b5] Harris SR, Clarke IN, Seth-Smith HM (2012). Whole-genome analysis of diverse *Chlamydia trachomatis* strains identifies phylogenetic relationships masked by current clinical typing. Nat Genet.

[b6] Jeffrey BM, Suchland RJ, Eriksen SG, Sandoz KM, Rockey DD (2013). Genomic and phenotypic characterization of *in vitro*-generated *Chlamydia trachomatis* recombinants. BMC Microbiol.

[b7] Kari L, Whitmire WM, Olivares-Zavaleta N (2011). A live-attenuated chlamydial vaccine protects against trachoma in nonhuman primates. J Exp Med.

[b8] Li Z, Chen D, Zhong Y, Wang S, Zhong G (2008). The chlamydial plasmid-encoded protein pgp3 is secreted into the cytosol of *Chlamydia*-infected cells. Infect Immun.

[b9] Matsumoto A, Izutsu H, Miyashita N, Ohuchi M (1998). Plaque formation by and plaque cloning of *Chlamydia trachomatis* biovar trachoma. J Clin Microbiol.

[b10] Miyairi I, Ramsey KH, Patton DL (2010). Duration of untreated chlamydial genital infection and factors associated with clearance: review of animal studies. J Infect Dis.

[b11] O'Connell CM, Nicks KM (2006). A plasmid-cured *Chlamydia muridarum* strain displays altered plaque morphology and reduced infectivity in cell culture. Microbiology.

[b12] O'Connell CM, Abdelrahman YM, Green E, Darville HK, Saira K, Smith B, Darville T, Scurlock AM, Meyer CR, Belland RJ (2011). Toll-like receptor 2 activation by *Chlamydia trachomatis* is plasmid dependent, and plasmid-responsive chromosomal loci are coordinately regulated in response to glucose limitation by *C. trachomatis* but not by *C. muridarum*. Infect Immun.

[b13] Peterson EM, Markoff BA, Schachter J, de la Maza LM (1990). The 7.5-kb plasmid present in *Chlamydia trachomatis* is not essential for the growth of this microorganism. Plasmid.

[b14] Ricci S, Ratti G, Scarlato V (1995). Transcriptional regulation in the *Chlamydia trachomatis* pCT plasmid. Gene.

[b15] Russell M, Darville T, Chandra-Kuntal K, Smith B, Andrews CW, O'Connell CM (2011). Infectivity acts as *in vivo* selection for maintenance of the chlamydial cryptic plasmid. Infect Immun.

[b16] Sachse K, Laroucau K, Riege K (2014). Evidence for the existence of two new members of the family *Chlamydiaceae* and proposal of *Chlamydia avium* sp. nov. and *Chlamydia gallinacea* sp. nov. Syst Appl Microbiol.

[b17] Seth-Smith HMB, Harris SR, Persson K (2009). Co-evolution of genomes and plasmids within *Chlamydia trachomatis* and the emergence in Sweden of a new variant strain. BMC Genomics.

[b18] Sigar IM, Schripsema JH, Wang Y (2014). Plasmid deficiency in urogenital isolates of *Chlamydia trachomatis* reduces infectivity and virulence in a mouse model. Pathog Dis.

[b19] Song L, Carlson JH, Whitmire WM (2013). *Chlamydia trachomatis* plasmid-encoded Pgp4 is a transcriptional regulator of virulence-associated genes. Infect Immun.

[b20] Song L, Carlson JH, Zhou B, Virtaneva K, Whitmire WM, Sturdevant GL, Porcella SF, McClarty G, Caldwell HD (2014). Plasmid-mediated transformation tropism of chlamydial biovars. Pathog Dis.

[b21] Stephens RS, Myers G, Eppinger M, Bavoil PM (2009). Divergence without difference: phylogenetics and taxonomy of *Chlamydia* resolved. FEMS Immunol Med Microbiol.

[b22] Stothard DR, Williams JA, Van der Pol B, Jones RB (1998). Identification of a *Chlamydia trachomatis* serovar E urogenital isolate which lacks the cryptic plasmid. Infect Immun.

[b23] Thomas NS, Lusher M, Storey CC, Clarke IN (1997). Plasmid diversity in *Chlamydia*. Microbiology.

[b24] Thylefors B, Negrel AD, Pararajasegaram R, Dadzie KY (1995). Global data on blindness. Bull World Health Organ.

[b25] Wang Y, Kahane S, Cutcliffe LT, Skilton RJ, Lambden PR, Clarke IN (2011). Development of a transformation system for *Chlamydia trachomatis*: restoration of glycogen biosynthesis by acquisition of a plasmid shuttle vector. PLoS Pathog.

[b26] Wang Y, Cutcliffe LT, Skilton RJ, Persson K, Bjartling C, Clarke IN (2013a). Transformation of a plasmid-free, genital tract isolate of *Chlamydia trachomatis* with a plasmid vector carrying a deletion in CDS6 revealed that this gene regulates inclusion phenotype. Pathog Dis.

[b27] Wang Y, Kahane S, Cutcliffe LT, Skilton RJ, Lambden PR, Persson K, Bjartling C, Clarke IN (2013b). Genetic transformation of a clinical (genital tract), plasmid-free isolate of *Chlamydia trachomatis*: engineering the plasmid as a cloning vector. PLoS ONE.

[b28] Ward ME (1983). Chlamydial classification, development and structure. Br Med Bull.

[b29] Xu S, Battaglia L, Bao X, Fan H (2013). Chloramphenicol acetyltransferase as a selection marker for chlamydial transformation. BMC Res Notes.

